# Cystectomy with orthotopic ileal neobladder reconstruction for treatment of bladder contracture after intravesical bacillus Calmette-Guerin therapy

**DOI:** 10.1590/S1679-45082014RC2794

**Published:** 2014

**Authors:** José Eduardo Vetorazzo, Leandro Augusto Costa Bahia, Bruno César Vedovato, Paulo Eduardo Goulart Maron, Paulo Ebert Esteves, Roni de Carvalho Fernandes, Marjo Deninson Cardenuto Perez

**Affiliations:** 1 Faculdade de Ciências Médicas, Irmandade da Santa Casa de Misericórdia de São Paulo, São Paulo, SP, Brazil.

**Keywords:** Urinary bladder neoplasms, BCG vaccine, Administration, intravesical, Cystectomy, Case reports

## Abstract

Bladder cancer is an important health problem worldwide due to high prevalence rates and costs related to treatment. A reduction in recurrence rates has been observed since the introduction of adjuvant intravesical immunotherapy with bacillus Calmette-Guerin. There are mild complications that are easily solved by local measures and orientations. Bladder contracture, a rare and severe local complication, in some cases leading to disability, is observed primarily in patients in a maintenance program. In this article we reported the case of a male patient who underwent transurethral resection of the bladder because of a high-grade T1 urothelial carcinoma and developed this complication during treatment with bacillus Calmette-Guerin. For this reason he was submitted to cystoprostatectomy with orthotopic ileal neobladder reconstruction.

## INTRODUCTION

Bladder cancer is an important health problem worldwide due to high prevalence rates and costs related to treatment.^(1)^ According to the *Instituto Nacional de Câncer* (INCA), approximately nine thousand cases were expected to occur in Brazil, in 2012. Out of these cases, 75% to 85% comprise superficial tumors, that is, the disease is confined to the mucosa (Ta-CIS) or submucosa (T1), and these patients are potential candidates for complementing treatment with chemotherapy or immunotherapy.^(1-3)^ The recurrence rates have dropped since the adjuvant intravesical immunotherapy with bacillus of Calmette-Guérin (BCG) was introduced.^(3)^

Although the use of BCG is considered effective, not all individuals benefit from it. Toxicity, diverse complications and no therapeutic response should be considered when proposing this adjuvant therapy.(^2^)

The main complications are mild and easily solved by local measures and orientations. Bladder contracture is a rare and severe local complication that leads to disability in some cases, and is observed primarily in patients in a maintenance program.^(4)^

## CASE REPORT

**Figure 1:**
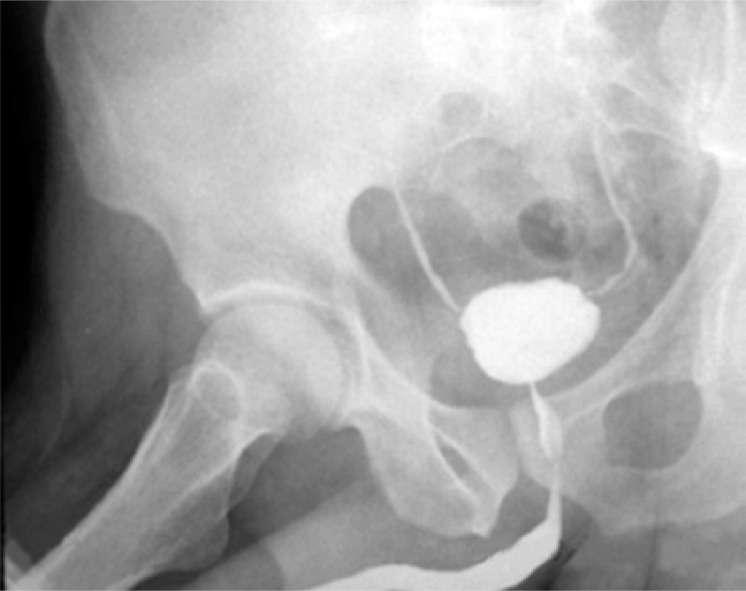
Urethrocystography. Small capacity bladder, bilateral grade I vesicoureteral reflux and post-micturition residual urine

**Figure 2:**
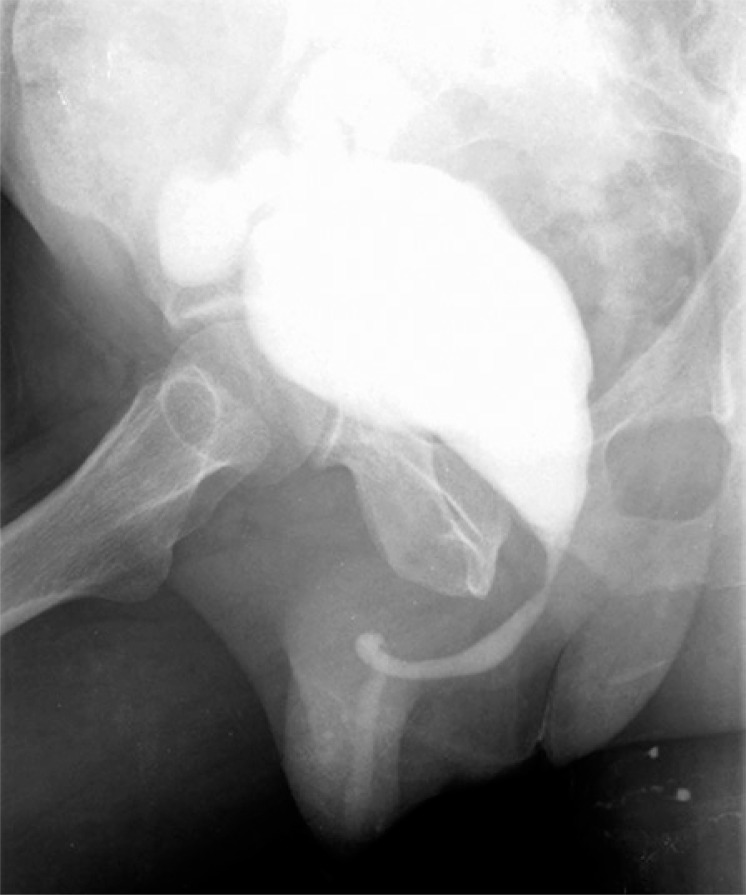
Urethrocystography. Neobladder in late postoperative period

A 53-year-old male smoker patient presented macroscopic hematuria with no pain for three days, in 2009. He went to the Emergency Department of the *Irmandade da Santa Casa de Misericórdia de São Paulo*, was submitted to ultrasound of the urinary tract that revealed an intravesical mass suggesting neoplasm in the left lateral wall. Transurethral resection (TUR) of the bladder was performed and he was diagnosed as high-grade T1 urothelial carcinoma. Eight weeks later the patient underwent another TUR but he had no recurrence or new lesions. Biopsies of the previously resected bed showed cystitis. He started on adjuvant intravesical therapy with BCG after one month and was treated for three years. According to the protocol of the organization, he was on induction phase for 8 weeks and on maintenance phase for 3 years (one weekly cycle for 3 weeks, at 3 and 6 months; and then every 6 months). In both phases he received an 80-mg dose in each session. In the second year of treatment the patient had no recurrences, and only some lower urinary tract symptoms (LUTS) related to storage, suck as pollakiuria and urgency, with no leakage. However, he felt adjusted to treatment and with no significant deterioration in quality of life. In the beginning of the third and last year of therapy, the urinary symptoms worsened and urinary frequency increased to 20 times a day, urgency with disabling incontinence and suprapubic pain with little bladder filling. In this period, it was necessary to discontinue treatment. Urethrocystography showed a small-capacity bladder, bilateral grade I vesicoureteral reflux and considerable post-micturition residual urine (Figure 1). The urodynamic study demonstrated very low vesical capacity (40ml) with enhanced sensitivity in this volume, besides high detrusor pressure (Pdet=78cmH_2_O), confirming the finding of bladder contracture. Cystoscopy during this period showed no neoplastic lesions. The patient underwent cystoprostatectomy due to incapacitating symptoms, with orthotopic ileal neobladder reconstruction and lymphadenectomy. A small bladder, with no other significant features, was observed during surgery. The procedure went uneventfully. The patient progressed with a urinary fistula in the ileal loop on the 10^th^ postoperative day, which resolved with conservative measures. He was discharged from hospital with no further events and followed up at the outpatient clinic for distension of the neobladder, with no complaints (Figure 2). The pathological examination revealed only inflammatory cystitis in the surgical specimen and absence of neoplasms.

## DISCUSSION

BCG is a first line therapy for high-grade Ta/T1 urothelial carcinoma and carcinoma *in situ* after TUR, particularly as maintenance therapy. It showed to be superior to intravesical chemotherapy in delaying tumor progression.^(1-3)^ However, it should not be administered immediately after resection; in general, one should wait for two weeks for local healing in order to avoid absorption and hematogenous dissemination of mycobacteria.^(1)^

Although there is no consensus about the ideal dose, six to eight instillations, once a week, followed by three maintenance doses every 3 to 6 months, in the first year, and every six months as from the second year, demonstrated promising results, according to the SWOG protocol, which was used in the present case.^(1)^

Assuming that maintenance therapy is necessary for optimal efficacy, toxicity becomes more relevant.^(2)^ It is substantially greater with intensive treatment, but severe secondary effects have been observed after few instillations.^(4)^ Storage symptoms (dysuria, pollakiuria and hematuria) are commonly associated side effects that occur usually after the third administration; they result from immune stimulation, with release of lymphokines as an associated inflammatory response.^(1)^

It is recommended to delay new instillations of BCG after several TUR and initiate anti-tuberculosis antibiotics, since they may be useful in cases of continued inflammation or infection.^(1,4)^

Bladder contracture is an uncommon complication but severe, and occurs in less than 1% of the patients.^(1)^ A series updated by Lamm et al. reported bladder contracture in only 5 (0.2%) out of of 2,602 patients evaluated.^(5)^

Nieder et al. identified only two cases of this complication out of 2,255 in two organizations, leading to a rate of 0.05 to 0.3%. In their study, both cases presented intravesical therapy complication as early as in the induction phase. One of these patients had evidence of bladder contracture after the fifth dose. There was probably leakage in the non-healed resection site, which would initiate an extensive perivesical inflammation and a fistula, resulting in bladder contracture.^(6)^

For this reason, such complication should be suspected in patients with intense micturition symptoms in any phase and, primarily in the maintenance phase of BCG immunotherapy.^(3,4)^ Some centers currently use prophylactic isoniazid in an attempt to decrease the incidence of contracture but the results vary.^(1,3)^ The use of associated interferon was also proposed to try to reduce the BCG dose and intolerance, and some series of few patients presented favorable results. The study conducted by Nieder et al.^(6)^ described the first case of bladder contracture in a patient on this combined therapy. Conservative treatment can be initially tried with hydrodistension and use of antimuscarinics.^(3)^ However, surgery should be considered in patients refractory to treatment or with incapacitating symptoms. Some options, such as bladder augmentation and cystectomy with reconstruction of the ileal conduit, might lead to tumor recurrence and negative effects on quality of life (QoL), respectively.^(6)^ Cystoprostatectomy with reconstruction of intestinal neobladder plays it role in improving QoL and being safer as to possible tumor recurrences.^(6)^

## CONCLUSION

Bladder contracture is a rare complication that must be thought of in patients with severe lower urinary tract symptoms and on Bacillus Calmette-Guérin therapy. When medical therapy is not possible, surgical approach with cystoprostatectomy and neobladder is the best option, for being a definite treatment and providing improved quality of life.
