# Supplementary material for: Cystectomy with orthotopic ileal neobladder reconstruction for treatment of bladder contracture after intravesical bacillus Calmette-Guerin therapy
Source: Einstein (Sao Paulo). 2014 Oct-Dec;12(4):502–4. doi: 10.1590/S1679-45082014RC2794 (PMC4879920; doi:10.1590/S1679-45082014RC2794)
Supplement: Translation

# Translation: Cistectomia com reconstrução de neobexiga ortotópica ileal para tratamento de bexiga contraída após aplicação de bacilo de Calmette-Guérin intravesical

**Authors:** José Eduardo Vetorazzo, Leandro Augusto Costa Bahia, Bruno César Vedovato, Paulo Eduardo Goulart Maron, Paulo Ebert Esteves, Roni de Carvalho Fernandes, Marjo Deninson Cardenuto Perez

**Affiliations:** 1 Faculdade de Ciências Médicas, Irmandade da Santa Casa de Misericórdia de São Paulo, São Paulo, SP, Brasil.

**Keywords:** Neoplasias da bexiga urinária, Vacina BCG, Administração intravesical, Cistectomia, Relatos de casos

## Abstract

O câncer de bexiga é um importante problema de saúde mundial, tanto pelas elevadas taxas de prevalência, quanto pelos custos relacionados ao tratamento. Desde a introdução da imunoterapia intravesical adjuvante com bacilo Calmette-Guérin, vem sendo observada diminuição na taxa de recorrência. As principais complicações são de pequeno porte e simples resolução a partir de medidas locais e orientações. A bexiga contraída, uma complicação local rara e grave, mas incapacitante em alguns casos, é observada principalmente em doentes com um programa de manutenção. Relatamos aqui o caso de um paciente masculino submetido a ressecção transuretral da bexiga por um carcinoma urotelial T1 de alto grau, que desenvolveu tal complicação durante tratamento com bacilo Calmette-Guérin, sendo portanto submetido à cistoprostatectomia com realização de neobexiga ortotópica ileal.

## INTRODUÇÃO

O câncer de bexiga é um importante problema de saúde mundial, tanto pelas elevadas taxas de prevalência quanto pelos custos relacionados ao tratamento.^(1) ^Segundo o Instituto Nacional de Câncer (INCA), aproximadamente 9.000 casos eram esperados para o ano de 2012 no Brasil. Desses casos, 75% a 85% são compostos por tumores superficiais, ou seja, doença confinada à mucosa (Ta-CIS) ou submucosa (T1), tornando esses pacientes potenciais candidatos para realização de terapia complementar com quimioterapia ou imunoterapia.^(1-3)^ Desde a introdução da imunoterapia intravesical adjuvante com bacilo de Calmette-Guérin (BCG), foi observada diminuição na taxa de recorrência.^(3)^

Embora o uso de BCG seja considerado muito eficaz, nem todos os casos têm benefício. Toxicidade, complicações das mais variadas e não resposta terapêutica devem ser levados em conta quando se propõe a adjuvância.^(2)^

As principais complicações são de pequeno porte, de resolução simples a partir de medidas locais e orientações. A bexiga contraída, uma complicação local rara e grave, incapacitante em alguns casos, é observada principalmente em doentes com um programa de manutenção.^(4)^

## RELATO DE CASO

Paciente do sexo masculino, 53 anos, tabagista, apresentou hematúria macroscópica indolor, de início há 3 dias, no ano de 2009. Procurou o Pronto-Socorro da Irmandade da Santa Casa de Misericórdia de São Paulo, sendo submetido à ultrassonografia do aparelho urinário, a qual revelou tumoração intravesical sugestiva de neoplasia em parede lateral esquerda. Foi realizada ressecção transuretral (RTU) da bexiga com diagnóstico de carcinoma urotelial T1 de alto grau. Após 8 semanas, foi submetido a nova RTU, dessa vez sem recidiva ou novas lesões. Biópsias do leito ressecado previamente revelaram cistite. Iniciou terapia adjuvante intravesical com BCG após 1 mês, com duração total de 3 anos. Conforme protocolo do serviço, realizou fase de indução por 8 semanas e de manutenção por 3 anos (1 ciclo semanal por 3 semanas, em 3 e 6 meses; a partir daí, a cada 6 meses), ambas com dose de 80mg em cada sessão. No segundo ano de tratamento, apresentava-se sem recidivas, com alguns sintomas do trato urinário inferior relacionados ao armazenamento, como polaciúria e urgência miccional sem perdas. Apesar disso, estava adaptado e sem deterioração significativa da qualidade de vida. No início do terceiro e último ano, os sintomas urinários pioraram, dessa vez com aumento da frequência urinária em torno de 20 vezes ao dia, urgência com perdas incapacitante e dor suprapúbica ao menor enchimento vesical. Nesse período, foi necessário interromper o tratamento. Uretrocistografia revelou bexiga de pequena capacidade, refluxo vesicureteral bilateral grau I e resíduo pós-miccional considerável (Figura 1). Estudo urodinâmico evidenciou capacidade vesical muito baixa (40mL) com sensibilidade muito aumentada nesse volume, além de alta pressão detrusora (Pdet=78cmH_2_O), confirmando o achado de bexiga contraída. Cistoscopia desse período não evidenciou lesões neoplásicas. Devido aos sintomas incapacitantes, o paciente foi submetido à cistoprostatectomia com realização de neobexiga ortotópica ileal e linfadenectomia. No intraoperatório, foi evidenciada bexiga de pequeno tamanho, sem outras características. Procedimento sem intercorrências. Evoluiu com fístula urinária da alça ileal no 10º pós-operatório, tendo resolução a partir de medidas conservadoras. Sem outras intercorrências, recebeu alta hospitalar e seguiu em acompanhamento ambulatorial sem queixas, em programa de distensão da neobexiga (Figura 2). O anatomopatológico revelou apenas cistite inflamatória na peça cirúrgica e ausência de neoplasia.

**Figura 1:** Uretrocistografia. Bexiga de pequena capacidade, refluxo vesicureteral bilateral grau I e resíduo pós-miccional

**Figura 2:** Uretrocistografia. Neobexiga em pós-operatório tardio

## DISCUSSÃO

O uso da BCG é considerado de escolha para carcinoma urotelial Ta/T1 de alto grau e carcinoma *in situ* após RTU, em particular a terapia de manutenção, a qual demonstrou ser superior à quimioterapia intravesical, no que diz respeito ao retardo da progressão tumoral.^(1-3)^ Entretanto, não deve ser administrada imediatamente após a ressecção, esperando-se, em geral, 2 semanas para a cicatrização local e evitando-se, assim, a absorção e a disseminação hematogênica das micobactérias.^(1)^

Embora não seja consenso a dose ideal, seis a oito instilações, uma vez por semana, seguidas por três doses de manutenção a cada 3/6 meses, no primeiro ano, e a cada 6 meses a partir do segundo ano, demonstraram resultados promissores, conforme o protocolo do estudo SWOG, o qual foi utilizado no caso em questão.^(1)^

Assumindo-se que a terapia de manutenção é necessária para eficácia ótima, a questão da toxicidade torna-se mais relevante.^(2)^ Esta é substancialmente maior com um tratamento intensivo, mas os efeitos secundários graves têm sido também observados depois de poucas instilações.^(4)^ Sintomas de armazenamento (disúria, polaciúria e hematúria) são efeitos colaterais comumente associados, ocorrendo geralmente após a terceira aplicação; trata-se de uma consequência da estimulação imunitária, com liberação de linfocinas como uma resposta inflamatória associada.^(1)^

O retardo de novas instilações de BCG após várias RTU e o início de antibióticos antituberculose é recomendado, pois podem ser úteis se inflamação contínua ou infecção estão presentes.^(1,4)^

A bexiga contraída, complicação incomum, porém uma das mais graves, ocorre em menos de 1% dos doentes.^(1)^ Uma série atualizada por Lamm et al. relatou bexigas contraídas em apenas 5 pacientes (0,2%) de 2.602 analisados.^(5)^

No trabalho de Nieder et al., foram identificados apenas dois casos dessa complicação num total de 2.255 em duas instituições, numa taxa de 0,05 a 0,3%. Neste estudo, ambos os casos apresentaram complicação da terapia intravesical ainda no esquema de indução, inclusive um deles com evidência de bexiga contraída após a quinta dose. Possivelmente, há extravasamento em um local de ressecção não cicatrizado, que iniciaria uma inflamação extensa perivesical e um trajeto fistuloso, resultando na bexiga contraída.^(6)^

Devido a isso, tal complicação deve ser suspeitada em pacientes com sintomas miccionais intensos em qualquer fase e, principalmente, na manutenção da imunoterapia com BCG.^(3,4)^ Atualmente, alguns centros utilizam isoniazida profilática na tentativa de diminuir sua incidência, porém com resultados variáveis.^(1,3)^ O uso de interferon associado foi proposto também na tentativa de diminuir a dose e a intolerância da BCG, com algumas séries de poucos pacientes apresentando resultados favoráveis. No estudo aqui citado foi descrito o primeiro caso de bexiga contraída em paciente na vigência dessa terapia combinada.^(6)^ O tratamento inicial pode ser tentado com medidas conservadoras, como hidrodistensão e uso de antimuscarínicos.^(3)^ Entretanto, naqueles refratários ou com sintomas incapacitantes, a indicação cirúrgica deve ser considerada. Opções como ampliação vesical e cistectomia com reconstrução de conduto ileal poderiam levar ao risco de recidiva tumoral futura e a efeitos negativos na qualidade de vida (QV), respectivamente.^(6)^ Já a cistoprostatectomia com reconstrução de neobexiga intestinal tem seu papel com melhora da QV e segurança quanto a possíveis recidivas tumorais.^(6)^

## CONCLUSÃO

A bexiga contraída é uma complicação rara mas que deve ser lembrada em pacientes com sintomas do trato urinário inferior grave em vigência da terapia com bacilo de Calmette-Guérin. Na intratabilidade clínica, a correção cirúrgica com cistoprostatectomia e neobexiga parece ser a melhor opção, devido ao seu tratamento definitivo e à melhora na qualidade de vida.
